# Priorities and opportunities for strengthening hygiene and One Health alignment in research, policy and practice

**DOI:** 10.1186/s44263-026-00337-z

**Published:** 2026-10-15

**Authors:** Max T. Eyre, Elizabeth D. Thomas, Fanta D. Gutema, Hongying Li, Kelly Baker

**Affiliations:** 1 Environmental Health Group, London School of Hygiene & Tropical Medicine, London, UK; 2 Department of Health Sciences, William & Mary, Williamsburg, Virginia USA; 3 Department of International Health, Johns Hopkins Bloomberg School of Public Health, Baltimore, Maryland USA; 4 Epidemiology, Biostatistics, Environmental Health, School of Public Health, The University of Memphis, Memphis, Tennessee USA; 5 Columbia Climate School, Columbia University, New York, USA; 6 Department of Epidemiology and Environmental Health, University at Buffalo State University of New York, Buffalo, New York, USA

**Keywords:** One Health, Hygiene, Transdisciplinary

## Abstract

Hygiene plays a critical yet under-recognized role in addressing One Health (OH) challenges, including zoonotic disease prevention, antimicrobial resistance (AMR) control, food safety and protection of ecosystem health. Likewise, hygiene research and interventionsgenerally lack an OH perspective, resulting in siloed efforts that inadequately address the human–animal–environment interface. We convened three sessions at global conferences well attended by health professionals working in hygiene or OH. Participants identified gaps and opportunities for hygiene-OH alignment across research, policy and practice at community, national, regional and global levels. An evidence gap identified was research on hygiene approaches to protect ecosystems in programs otherwise focused upon human or animal health. Methodological priorities included harmonizing tools and protocols for pathogen detection, fecal contamination measurement and hygiene behavior assessment; strengthening cross-sectoral data sharing; and developing integrated study designs. Intervention opportunities included engaging local stakeholders, balancing infrastructure and behavior change, adapting existing programs for cross-sectoral relevance and broadening evaluation to include animal and environmental outcomes. Implementation priorities included localized program design and decision-making, regional cooperation for cross-border threats and leveraging surveillance and preparedness infrastructure. Policy priorities included reducing institutional fragmentation, harmonizing standards, and strengthening the roles of hygiene and OH within existing regulatory frameworks. From our synthesis, we present cross-cutting recommendations for strengthening hygiene and OH alignment. Aligning hygiene and OH offers a pathway to more comprehensive and upstream prevention of disease and AMR, supporting coordinated, system-level management of health risks across human, animal and environmental domains and building resilience to emerging health threats.

## Background

### Why strengthen hygiene and One Health alignment?

One Health (OH) is a collaborative, multisectoral and systemic approach that recognizes the interconnection between human, animal and ecosystem health [1, 2]. It promotes a sustainable and healthy future through collaboration, communication, capacity building, and coordinated action across public health, veterinary medicine, environmental public health, agriculture and related sectors to prevent, detect and respond to health threats [3, 4]. Major OH challenges include zoonoses and food safety [5–7], antimicrobial resistance (AMR) [8] and environmental issues driven by pollution, habitat loss and climate change [9, 10]. Addressing these challenges requires preventative and upstream measures and coordinated response across sectors, as articulated in the Quadripartite One Health Joint Plan of Action (OHJPA) for OH implementation (Food and Agriculture Organization, United Nations Environment Programme, World Health Organization and World Organization for Animal Health) [4].

Hygiene is defined as the practices and conditions that reduce or prevent exposure to pathogens and other health hazards, particularly through cleanliness [11]; hygiene operates at the human-animal-environment interface but is often under-recognized in OH approaches [12]. Hygiene practices aim to interrupt pathogen transmission pathways linking people, animals and ecosystems [13] and can include handwashing, safe management of human and animal waste, cleaning of shared living and working spaces (e.g., homes, farms, markets), environmental management (e.g. water management) and biosecurity measures [13]. Despite its essential role in OH implementation, hygiene is predominantly framed within human-oriented water, sanitation and hygiene (WASH) or infection prevention efforts [12, 14–17].

Failure to adequately align hygiene and OH can undermine the preventative potential of OH for emerging health risks by leaving critical human-animal-environment interfaces insufficiently addressed. When hygiene measures are not applied across biological species and shared environments, pathogens and other hazards can persist and circulate through pathways such as contaminated water, food systems and shared spaces, resulting in missed opportunities for upstream risk reduction [17, 18]. This gap shifts OH efforts toward downstream reactive responses that are often more costly and less sustainable than preventative approaches [19]. Fragmented governance, in which hygiene remains institutionally disconnected from animal and ecosystem health, reduces efficiency by creating parallel and uncoordinated measures and constrains the ability to address cross-sectoral risks. For example, hygiene-related risks in shared human–animal environments such as markets or food production settings may fall between sectoral mandates, leaving no single authority responsible for addressing the full transmission pathway. This challenge is particularly acute for hygiene, which cuts across multiple sectors and everyday practices and therefore risks being treated as everyone’s responsibility, but no one’s mandate, underscoring the need for a clearly defined institutional role within OH frameworks. Weak coordination also exacerbates implementation and equity gaps. Communities engaged in informal or resource-dependent livelihoods (e.g., wildlife farmers, hunters, communities reliant on natural resources) often face elevated exposure risks yet fall outside existing sectoral mandates, despite hygiene measures potentially offering effective and accessible preventive strategies [20, 21].

Improved alignment of hygiene and OH activities can generate co-benefits for humans, animals and ecosystems. For example, preventative hygiene interventions can enhance food safety and nutritional outcomes by reducing contamination along livestock and food value chains, while also supporting animal health and productivity and protecting workers [22, 23]. Environmental co-benefits include reduced contamination of water bodies and soil, protection of aquatic ecosystems and biodiversity, and the preservation of ecosystem services critical to livelihoods and climate resilience [24].

Although the OHJPA recognizes that insufficient coordination with WASH efforts undermines health outcomes for people, animals and ecosystems [4], it provides limited operational guidance on how hygiene can be systematically incorporated into OH implementation. At the same time, hygiene programs largely remain human health-focused and rarely adopt an OH perspective that considers animal and biodiversity perspectives [12]. In this perspective, we discuss priorities and opportunities for improved alignment between hygiene and OH actions, programs and sectors as a bidirectional exchange: applying OH multisectoral collaboration approaches within hygiene programming and strengthening the visibility and prioritization of hygiene strategies with co-benefits to health across species within OH policy and practice. Improved alignment has the potential to enhance cross-sectoral preventative capacity and improve resilience to emerging health threats [23, 25, 26]. In this context, we drew on multi-sectoral consultations with stakeholders working in OH and hygiene sectors to map gaps and priorities for improved hygiene and OH alignment across research, policy and practice at community, national, regional and global levels. Emergent themes where there was consensus informed our recommendations on opportunities for more coherent, preventative and cost-effective action to improve the shared health of humans, animals and ecosystems. All definitions and scope used in this perspective article are outlined in Table 1.

**Table 1:** Definitions and scope used in this perspective article

Term	Definition and scope
**One Health (OH)**	A collaborative, multisectoral approach that recognizes the interconnectedness of human, animal and ecosystem health and seeks coordinated action across sectors to prevent and respond to cross-cutting health threats [1, 2]. OH challenges include zoonotic disease and food safety, antimicrobial resistance and environmental issues driven by pollution, habitat loss and climate change. This usage aligns with definitions from the Quadripartite agencies and the One Health High-Level Expert Panel. There is no single, universally adopted One Health “framework” and we used ‘One Health approach’ as an umbrella term.
**Hygiene**	The practices and conditions that reduce or prevent exposure to pathogens and other health hazards, particularly through cleanliness [27]. Within a One Health framework, hygiene applies across humans, animals and shared environments, encompassing personal and community hygiene, animal care and biosecurity, and environmental sanitation and resource protection. Examples include handwashing; safe water management and food handling; sanitation and waste management; hygiene in livestock production, animal housing and markets; vector control; and practices that prevent contamination of air, soil and water.
**Ecosystem health**	The condition, resilience, and functioning of ecological systems that mediate interactions among humans, animals and pathogens [28–30].
**Environmental public health**	The study of how the natural, built and social environments influence human health and well-being [31–33].
**Environmental hygiene**	The management of environmental conditions and contamination pathways across water, waste, soil, shared surfaces and built environments to sustain ecosystem, human and animal health [34].
**Alignment of One Health and hygiene**	The purposeful prioritization and clearer specification of hygiene practices within OH research, interventions, implementation strategies and policy, and reciprocally, the application of OH perspectives within hygiene programming, with co-benefits for human, animal and ecosystem health

### Conference consultations

**Fig. 1:**
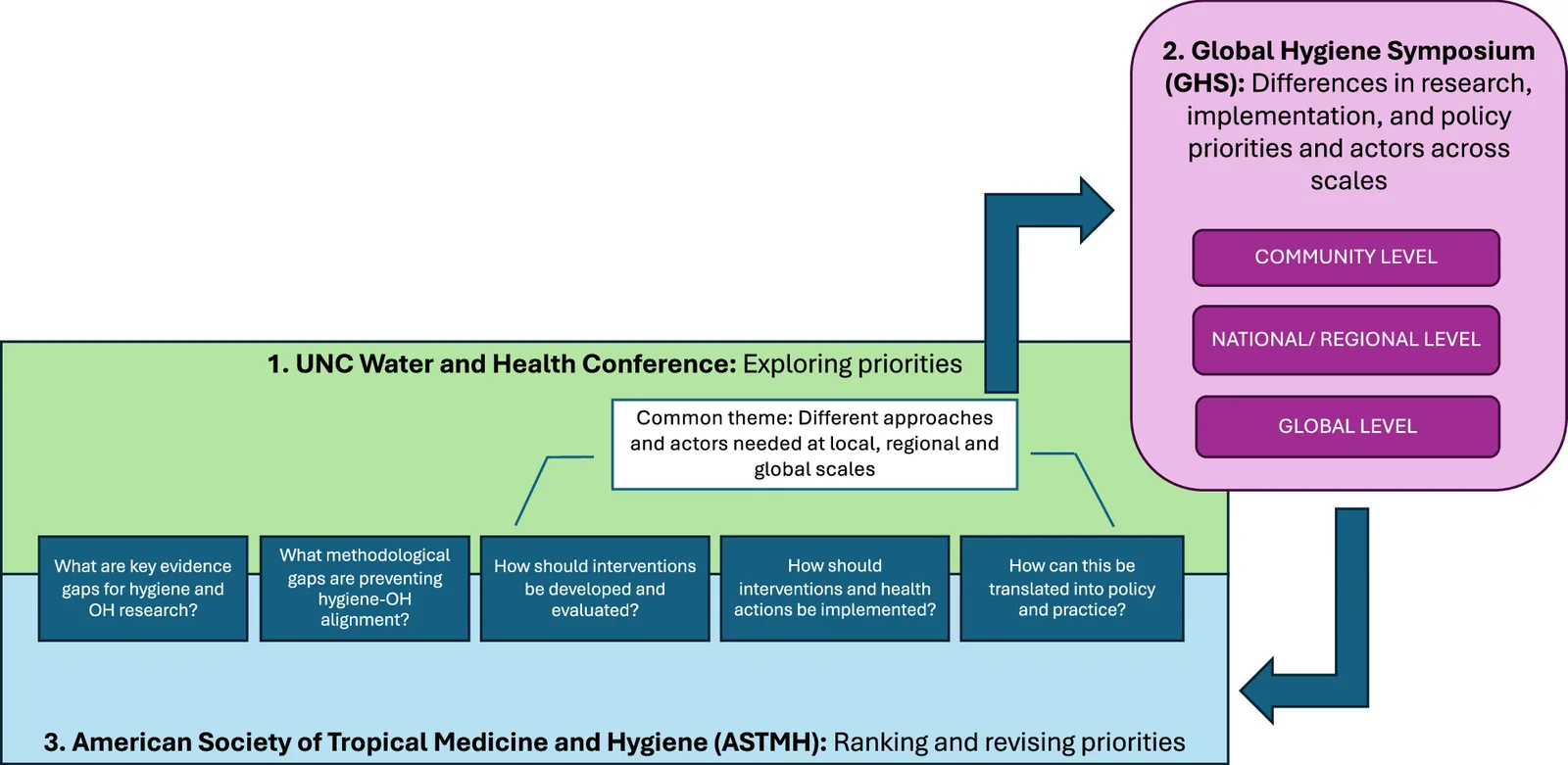
Structure and focus of the three conference sessions

To identify priority areas for OH and hygiene alignment, we convened a series of structured and interactive sessions at three international conferences: the 2023 University of North Carolina (UNC) Water and Health Conference (https://waterinstitute.unc.edu/our-work/unc-water-and-health-conference), the 2023 Global Hygiene Symposium (GHS) organized by the Reckitt Global Hygiene Institute and Chatham House and the 2024 American Society of Tropical Medicine and Hygiene (ASTMH) Annual Meeting (https://astmh.org/annual-meeting). These sessions were designed to elicit perspectives on evidence gaps, methodological challenges and strategic priorities for advancing alignment of hygiene and OH in research, policy and practice at community, national, regional and global levels (Fig. 1).

Participants in these sessions represented a broad range of disciplines and sectors, including epidemiology, environmental public health, WASH, veterinary/animal health, social science, policy and implementation from academia, government, non-governmental organizations and community practice across multiple geographic regions, including North and South America, Europe, East, West, Central and Southern Africa, South and Southeast Asia and Australia.

At UNC (October 2023), after a series of presentations on hygiene-related research projects with a One Health perspective, we hosted a World Café session [35] where participants were asked to reflect on five themes (research gaps, research methodology, intervention development, evaluation of interventions and programs and translation of research into practice) and discuss priorities and challenges for hygiene and OH alignment. At GHS (January 2024), we adapted this format and foregrounded concepts of stakeholders and scale, asking participants to identify priorities and actors at community, regional and global levels. Finally, building on insights from both events, we hosted an interactive symposium at ASTMH (November 2024) to share feedback from prior conferences and discuss priorities identified in the previous sessions.

This perspective article synthesizes and presents insights and reflections from the conference-based consultations to highlight current gaps and priorities for strengthening hygiene and OH alignment to improve human, animal and ecosystem health. The viewpoints shared here reflect the authors’ interpretations and reflections alongside the collective input of practitioners, researchers, policymakers and implementers who participated in the conference sessions.

## Gaps and priorities for strengthening hygiene and One Health alignment 

### Evidence gaps and priorities for research

Efforts to reduce zoonotic risks in hygiene and OH have traditionally focused on human and animal health, with limited attention to the environment [36]. Strategies often target human–animal interactions (disinfection, safe animal waste management, biosecurity, physical separation) and have been strengthened by growing WASH-OH alignment [12, 17], especially to reduce AMR and enteric pathogen exposure [15, 23, 37]. However, the environmental dimension remains under-addressed, despite its recognized importance as a determinant of ecosystem health and disease transmission, with the pathways mediated by environment less well understood than human–animal routes. AMR underscores the urgency of linking hygiene across human, animal and environmental systems that shape ecosystem health [38].

Across the three sessions, conference participants extensively discussed this need to strengthen research on the environmental component and have better coordination across the human-animal-environment interface. They linked this to several research priorities: (1) studying disease transmission and health across multiple species and whole ecosystems; (2) assessing costs and benefits of human–animal proximity; and (3) advancing hygiene’s role in tackling AMR. These priorities are explored throughout the rest of this section.

Conceptual clarity about “environment” is essential. The environment was formally introduced as a component of OH in 2008, with a new strategic framework that acknowledged the shared ecosystems in which humans and animals interact and upon which infectious diseases are transmitted [16]. Yet, in practice, the environmental component remains poorly defined and underprioritized in health programming [36]. It is often treated as an omnibus category, encompassing everything outside of human and veterinary medicine (e.g., physical/chemical properties, plants, algae, fungi). Conference discussions highlighted the need to move beyond viewing the environment as a passive fomite or taxonomic list of non-animal species and toward viewing it as a dynamic system mediating multispecies interactions and ecosystem health. This framing shapes hygiene practice. When the environment is viewed primarily as a transmission site, hygiene efforts focus on sterility and safety of food, water and surfaces used by humans and animals. Participants highlighted several evidence gaps within this framing including: (1) health risks for animals, and especially their needs for clean food and water; (2) the effects of seasonality, extreme weather (e.g., flooding) and climate change on human-animal-environment interactions, transmission within and between species, hygiene conditions, infrastructure and programs; and (3) WASH and OH research’s heavy focus on households while often neglecting important transmission pathways in peridomestic and public spaces, both in the built environment (e.g., airports, train stations, event spaces) and wild spaces (e.g., forests and wetlands) [12].

Alternatively, foregrounding ecosystem health can shift the emphasis from transmission prevention to sustainability-centered hygiene approaches that aim to protect multiple species from infectious agents and physical and chemical hazards. This conceptualization of the environment as an interconnected ecosystem is essential for understanding how it shapes disease dynamics both within and between species. At UNC and GHS, participants emphasized the need to measure environmental contaminants and detect pathogens across broader ecological contexts and species (including plants, aquatic systems and other macrobiotic species) and settings, like markets and schools.

Participants also noted differing framings of human-animal interactions across settings and sectors. For example, WASH practitioners and researchers, coming from a perspective traditionally focused on human disease risk reduction, may emphasize limiting close contact with domestic animals and livestock, while overlooking the potential social and economic benefits of those interactions [39, 40]. Participants called for broadening hygiene-OH research beyond handwashing and diarrheal disease to explore the links between hygiene practices and animal care and nutrition, and to address the balance between maintaining animal welfare and reducing zoonotic risk.

AMR was a consistent research priority across conference discussions. Participants highlighted the need for stronger evidence on how hygiene interventions across the animal-based food chain can reduce foodborne and zoonotic disease risks and interrupt AMR transmission pathways [23, 41]. Key gaps include understanding how hygiene, sanitation and animal husbandry practices influence antibiotic use in food production systems, and the extent to which improved hygiene can reduce reliance on antibiotics while supporting animal health and productivity.

A key question raised was whether hygiene can realistically substitute for antibiotics or vaccines in practice, with producers needing support and incentives to adopt hygiene-based strategies. From the authors’ viewpoint, while hygiene cannot replace these interventions, it can play complementary roles in disease prevention and control and indirectly contribute to the mitigation of AMR, especially in resource-limited settings and areas where access to veterinary care and healthcare is challenging. Within a One Health framework, hygiene functions as a cross-sectoral preventive measure that reduces pathogen transmission across human, animal and environmental systems and, by lowering infection incidence, may reduce demand for antibiotic use. Session participants emphasized the need for research evaluating the effectiveness, feasibility and incentives required to support adaptation of hygiene-based strategies in livestock production and food systems as part of a broader commitment to reducing AMR. They highlighted that this needed to take place alongside coordinated policies, infrastructure investments and surveillance systems to support AMR prevention.

Overall, there is a pressing need for hygiene-OH research to inform interventions that reduce current and emerging risks related to zoonotic (and zooanthroponotic [42]) disease transmission and AMR. This will require clearer and more purposeful definition and application of the environmental dimension across sectors [18].

### Methodological gaps

Recent years have seen considerable advancements in tools and systems relevant to hygiene and OH, alongside greater cross-sectoral communication, cooperation and information sharing. Innovations in diagnostic technologies, increased capacity for genomic analysis, expanded clinical, veterinary and agricultural disease surveillance networks and the emergence of wastewater epidemiology as a surveillance tool have strengthened our capacity to detect and respond to global health threats [43–45]. Despite this progress, significant methodological gaps remain, hindering our ability to fully understand and target the complex interactions among humans, animals and their shared environment that determine multispecies health.

A recurring theme across the three conference sessions was the difficulty of applying existing methods to transdisciplinary research, defined here as research in which study questions, data collection and analysis are jointly developed and integrated across multiple disciplines and sectors (e.g. public health, veterinary science, environmental public health and social science) rather than conducted separately within disciplinary silos [45]. One successful example is the joint collection and analysis of human and animal data for environmentally persistent zoonoses in urban Brazil [46]. Five key methodological gaps emerged from the session discussions: (1) the lack of standardized tools for measuring fecal contamination; (2) limited availability of integrated and affordable diagnostics for pathogen detection; (3) challenges in assessing human exposure to fecal contamination and measuring hygiene-related behaviors; (4) fragmented data systems; and (5) a need for integrated transdisciplinary study designs and new analytical tools. The remainder of this section explores each gap in turn.

Environmental hygiene monitoring tools for assessing fecal contamination are well established in regulated industries (e.g., food production) but are inconsistently applied and often unsuitable for other OH-relevant settings, such as households, farms, schools, markets and shared public spaces. These tools are typically designed for controlled environments with defined surfaces, standardized workflows, and relatively uniform contamination sources [47, 48], and are therefore likely to perform poorly in settings characterized by diverse contamination pathways, intermittent exposures and mixed human and animal sources [49]. Although many studies monitor fecal pathogens in food, water, soil and on surfaces, conference participants emphasized the need for standardized, accessible fecal contamination assessment tools that are usable by researchers, public health officers, veterinarians and environmental inspectors. They also stressed the need for methods that can distinguish human from animal contamination, where transmission pathways are unclear. Microbial source tracking (MST) uses genetic markers to distinguish between species and shows promise [50] but remains technically complex and difficult to scale in resource-constrained settings. While conference discussions centered around fecal contamination, there is also a need for environmental monitoring tools for environmentally transmitted diseases spread by other biological products, like skin or saliva.

A related gap is the limited availability of affordable, integrated diagnostic tools for comprehensive multi-pathogen surveillance across human, animal and environmental domains [45, 51]. Participants called for diagnostics suitable for laboratory and field use, especially in resource-limited settings, alongside investment in diagnostic and microbiological capacity in public health and veterinary sectors to enable routine, cross-sectoral surveillance.

Assessing human exposure to animal fecal contamination—an important transmission pathway at the human–animal–environment interface—adds another layer of complexity. Most studies rely on proxy survey indicators (e.g., animal ownership or proximity to livestock) [37], which do not capture the specific exposure pathways through which humans encounter animal fecal contamination, and therefore provide limited insight into the behaviors, environmental conditions, and management practices that shape transmission risk and that could be addressed through targeted interventions. Diverse and inconsistent methods are also commonly used, further limiting comparability across studies and settings [52]. Tools such as the fecal-oral child exposure to zoonotic enteropathogens index (FECEZ Enteropathogens Index) are beginning to address these gaps through validated, multi-dimensional, survey-based measures that capture self-reported behaviors and environmental risk observations across shared human–animal environments [53]. Strengthening such measurement approaches is essential for designing and evaluating hygiene interventions that operate across sectors within a One Health framework.

Additionally, accurate measurement of hygiene-related behaviors (e.g., handwashing or animal interactions) can be hampered by social desirability bias [54, 55] and constraints associated with structured observation (e.g., acceptability of observing private routines, short observation periods missing key events, and the high time and cost required) [56]. Participants recommended addressing this by triangulating data, such as combining surveys with structured direct observations, geo-tracking and activity diaries. Qualitative and mixed methods approaches were also highlighted for their ability to provide insights into contextual drivers of persistent risky behaviors, particularly in settings with high levels of health awareness [57]. Reducing stigma and reporting biases through innovative data collection strategies was identified as crucial for improving behavioral data validity.

Another major barrier identified was the fragmentation of data systems across sectors. Human, animal and environmental agencies often collect and store information separately using incompatible protocols and platforms. This siloed approach prevents analysis of shared exposure pathways, or assessment of cumulative effects of multiple hygiene-related risks across domains [58]. For example, this can make studying the links between the following challenging: agricultural runoff and human drinking-water contamination; farm waste management and community zoonotic risk; and zoonotic spillover through veterinary illness records and local gastrointestinal outbreaks. Participants stressed the need for shared digital platforms, standardized data collection methods and coordinated data-sharing across health, agriculture and environmental sectors to enable joint analysis and holistic risk assessment.

Transdisciplinary research is increasingly seen as essential for addressing OH problems [59] and this is also the case for hygiene-OH alignment. Conference discussions underscored the value of transdisciplinary approaches, such as eco-epidemiological studies that collect and integrate human, animal and environmental data within a single study design and food-chain risk modelling to analyze risks at the human-animal-environment interface and guide targeting of hygiene interventions. These designs require statistical methods that can model multiple, interconnected outcomes across domains and species. These analyses can be supported by the availability of high-resolution global datasets, including satellite-derived land-cover data (e.g., ESA WorldCover) [60], climate data (e.g., ERA5-Land) [61], and aggregated mobile-phone data for characterizing human mobility (e.g., approaches developed by WorldPop) [62].

To fully capture the complex drivers of pathogen transmission between species and fomites within an ecosystem, large-scale, mixed-methods longitudinal studies are essential. Integrated, multi-data-source designs, as illustrated by the PATHOME study in Kenya [63], demonstrate the value of combining longitudinal pathogen detection, detailed behavioral observation, environmental sampling and spatial analysis to document when, where, how and why pathogen exposures occur. Linking repeated measurements across human, animal and environmental domains to socioeconomic and infrastructural contexts can reveal critical hygiene intervention points and provide the data needed to build and test models that explore the potential effects of interventions. These multi-component study designs offer a richer and more nuanced understanding of pathogen transmission dynamics than can be achieved by single cross-sectional surveys. However, inclusion of biodiversity indices would strengthen understanding of the beneficial or harmful impact of interventions on ecosystem health [64]. Overall, closing these methodological gaps will require improved, more diverse tools and substantial investment in cross-sectoral training, infrastructure and capacity so they can be used effectively across human, animal and environmental domains. Progress will depend on stronger collaboration across disciplines and sectors, supported by mechanisms for harmonizing methodologies and data collection standards and systems across settings [18, 58].

### Intervention development and evaluation

Health-related interventions, whether targeting human, animal or ecosystem health, are heavily siloed—designed and evaluated in isolation using indicators and standards that are typically specific to a single disease or sector. For example, hygiene interventions aimed at reducing human exposure to fecal contamination in domestic settings rarely consider animal hygiene [12, 37]. This siloing is also reflected in shared targets for global health improvement, where the safe management of animal waste and water quality for animals is largely absent, despite their relevance to human, animal and ecosystem health [12]. This siloed nature of hygiene and OH interventions, noted repeatedly during conference discussions, reinforces the need for greater coordination, collaboration, and communication across the human-animal-environment interface, as outlined in Evidence gaps and priorities for research section.

Four themes emerged from these discussions: (1) engaging local stakeholders for culturally relevant, cross-sectoral interventions; (2) balancing infrastructure upgrades with behavior change while adapting existing interventions; (3) incorporating ecosystem restoration into hygiene-OH efforts; and (4) broadening evaluation to include diverse outcomes and improved cross-sectoral communication.

Local stakeholder engagement, including community leaders, veterinary and public health services, was identified as essential for ensuring interventions are culturally relevant and supported by the community. Generating buy-in from local leaders and organizations was seen as critical for sustaining policy and funding support. Participants suggested that adopting participatory approaches to intervention development and evaluation with cross-sectoral stakeholders would help to ensure that hygiene-OH interventions expand beyond a focus on human health. For example, interventions could also aim to support animal productivity and generate related economic gains.

Balancing infrastructure improvements and behavior change in hygiene interventions was another priority, acknowledging the tendency for WASH interventions to rely on individual behavior change in settings where services and infrastructure are not accessible. Household-level interventions that do not acknowledge structural challenges can place disproportionate burden on poor households by shifting responsibility from the state to individuals; they also risk reinforcing gender inequalities, given that women are frequently the household caregiver (human and animal) and hygiene manager, and therefore the target for behavior change interventions [65]. This must be considered carefully when designing hygiene-OH interventions [66]. Participants emphasized the importance of identifying interventions that require minimal behavior modification or participant burden, such as reorganizing existing practices to improve hygiene rather than introducing entirely new behaviors. Where possible, existing interventions should be adapted to incorporate human, animal and environmental components, rather than designing new interventions. For example, WASH programs could partner with veterinary services to integrate animal vaccinations and waste management into a single intervention, which was noted as a promising area for joint hygiene-OH programming [67].

Ecosystem restoration received little attention in conference discussions, yet several ecosystem-level interventions are directly relevant to hygiene-OH. Biological control of pests and weeds, for example, can reduce fecal contamination, pathogen spread, or human and animal contact with contaminated environments, with potential benefits across OH domains [68]. More broadly, nature-based solutions such as improved drainage, vegetation management in peridomestic areas and ecosystem-based flood control can support ecosystem health by protecting the environment from disruptive flooding events while reducing environmental contamination and interrupting exposure pathways across the human-animal-environment interface. These approaches are particularly relevant in resource-limited settings, where they can simultaneously support disease prevention, livelihoods, economic development and climate resilience.

In terms of intervention evaluation, participants across conferences highlighted the need to: (1) improve collaboration between public, veterinary and environmental public health sectors, to ensure evaluations are designed to assess impact across all three domains, as well as stakeholders across local, regional and national levels; (2) design studies to capture both short- and long-term impacts of interventions; and (3) broaden evaluation outcomes to include wellbeing and non-health outcomes, such as quality of life and animal productivity to support cost-benefit analyses.

In summary, hygiene-OH interventions and their evaluations require input from diverse local stakeholders across OH domains. Continued siloing of interventions and indicators of success will limit opportunities to combine existing intervention approaches across sectors, reducing the potential for more impactful and sustainable outcomes.

### Implementation strategies

Four priority areas were identified for implementation of hygiene-OH interventions and health actions: (1) tailoring interventions to local systems and culture; (2) fostering community engagement and local decision-making; (3) addressing cross-border risks with regional surveillance and response; and (4) strengthening global coordination by leveraging existing preparedness infrastructure and partnerships.

Translating hygiene-OH research into action requires context-specific and -sensitive, scalable strategies grounded in systems thinking, where interventions are holistic and address problems as part of a larger system rather than in isolation. Implementation science shows that the effectiveness of an intervention in one context does not guarantee success elsewhere; outcomes depend on local governance, culture, sociopolitical conditions, stakeholder engagement and resource constraints [69–71]. Across conferences, participants stressed that interventions must be adapted to setting-specific determinants and delivered through locally appropriate mechanisms.

Community engagement was identified as a key principle for successful implementation, building on the narrative in the *Intervention Development and Evaluation* section. Participants emphasized the importance of implementing interventions with local actors to enhance cultural relevance, ownership and sustainability [72]. Interventions were seen as more likely to succeed when aligned with existing cultural practices and when framed around tangible benefits, such as increased household productivity, rather than simply correcting hygiene “deficits.” Examples discussed included improving hygiene practices for animal husbandry by explaining their impact on animal productivity to communities. Localization of both delivery and decision making was another priority for implementation. For example, USAID’s former localization strategy aimed to redirect power and funding toward local institutions, prioritizing community ownership, co-design and local knowledge [73]. Conference discussions reinforced the need to shift decision-making power to institutions in low- and middle-income countries (LMICs) to ensure equity and long-term impact, especially in resource allocation, priority setting and identification of relevant local actors. However, it should be noted that in many countries, well-designed and contextually appropriate hygiene and OH policies can still remain unimplemented due to chronic constraints in financial, technical and human resources, highlighting a persistent barrier between policy ambition and delivery capacity [74, 75].

At the regional level, conference participants stressed the importance of addressing cross-border risks, especially for zoonoses and AMR, driven by human and animal movement and ecosystem disruption [76, 77]. Strengthening regional surveillance systems can improve data sharing, harmonize laboratory capacity and support coordinated outbreak response networks [78], including linking AMR surveillance across human and animal sectors and regions using shared protocols and platforms. Expanding environmental microbiology capacity, especially in regional hubs, was also identified as critical for early detection and response.

Global-level implementation demands coordination between sectors and international actors, particularly in the wake of COVID-19. Pandemic preparedness increasingly calls for investments that support integrated OH surveillance and response, incorporating environmental monitoring, data sharing and institutional partnerships. Participants discussed leveraging existing pandemic preparedness infrastructure, such as genomics labs or wastewater testing platforms, to support broader hygiene surveillance, alongside building global cross-sector coordination across environment, health, agriculture and veterinary sectors for a cohesive OH approach.

### Policy development

Strengthening alignment between hygiene and OH within policy frameworks is a complex and evolving challenge. Hygiene is addressed across sectors through existing policy domains, including public health, WASH, food safety, and agriculture, but often in fragmented and siloed ways. Global initiatives such as the Sustainable Development Goals (SDGs) have codified hygiene as a health priority, particularly through indicators related to hand hygiene and sanitation [79]. However, while hygiene is embedded in SDG 6 (Clean Water and Sanitation) and elements of animal and ecosystem health appear in SDGs 2 (Zero Hunger), 3 (Good Health and Well-being), 12 (Responsible Consumption and Production), 13 (Climate Action) and 15 (Life on Land), the SDGs do not explicitly address interconnections between human, animal and ecosystem health. This limits visibility and accountability for hygiene-OH policy actions, particularly in relation to upstream environmental exposures and animal waste management.

Five main priority areas for policy development were identified in discussions: (1) reducing fragmentation and inconsistencies in hygiene policy; (2) harmonizing standards and enforcement across sectors; (3) aligning regional and national policies; (4) embedding hygiene-OH goals into existing frameworks; and (5) building public awareness to support adoption and compliance.

At the global level, participants highlighted inconsistencies in hygiene-related policies across countries and sectors. For example, hygiene policy for live animal and food markets varies widely between countries, with calls for global monitoring of infrastructure such as toilets, handwashing facilities and separation of slaughter areas from produce [80, 81]. As highlighted during the ASTMH session, veterinary access to antibiotics is highly restricted in some regions (e.g. parts of India and Uganda), while human access through unregulated pharmaceutical markets remains widespread, leading to cross-use in animals [82]. Such mismatches can contribute to AMR and compromise hygiene-related infection control efforts, underscoring the need for harmonized regulation within and between countries and across supply chains. In agreement with the narrative on localization in the *Implementation Strategies* section, several participants noted that while international treaties and guidelines can promote best practices, policy implementation must be locally adapted to infrastructure capacity and the sociopolitical context.

Sector-specific policies govern hygiene in food production, animal husbandry and healthcare, but enforcement varies widely by setting [83–85]. For instance, while hygiene standards are well-defined and regulated in formal food processing, upstream domains such as smallholder farming or confined animal feeding operations often lack enforceable standards or adequate monitoring. A related gap is the limited regulation of environmental pathways, such as untreated animal waste discharge and contaminated water and soil systems, despite their role in zoonotic spillover and AMR transmission [86]. Participants stressed that effective hygiene-OH policy must be cross-sectoral and multilevel, linking community bylaws, national legislation, regional coordination and international frameworks to ensure coherence across risk pathways rather than isolated sectoral compliance.

While global strategies such as the Quadripartite OH Joint Plan of Action [4] provide strategic direction, participants noted that operationalizing these frameworks is often hindered by fragmented governance, weak enforcement and limited intersectoral coordination. Guidance for aligning OH and WASH in policy remains vague. AMR was repeatedly cited as both a critical challenge, for which multi-country coordination and policy action are urgently needed yet inconsistently implemented, and an opportunity to embed hygiene into OH approaches, as hygiene is central to AMR prevention in healthcare and animal husbandry [4, 87]. At the national level, many AMR National Action Plans (NAPs) that are explicitly grounded in One Health offer a strategic entry point for institutionalized hygiene within cross-sectoral governance. While AMR NAPs often address infection prevention and control, biosecurity and antimicrobial stewardship, hygiene at shared human–animal–environment interfaces remains unevenly operationalized [88]. Better incorporation of hygiene within NAP implementation, particularly across environmental management, food value chains and informal production systems, could enhance bidirectional engagement between AMR and broader OH agendas [89].

At the regional level, harmonization of hygiene-related policies was identified as a priority. Participants emphasized the importance of establishing regional hygiene standards, aligning AMR control measures and coordinating zoonotic disease prevention frameworks across borders to avoid contradictory policies and support coherent action. Local regulation is critical to the success of hygiene-OH policy. Culturally aligned hygiene regulations developed with input at the community-level, such as animal care practices or waste management protocols, can improve compliance [90]. However, participants noted that development and implementation of policy at municipal scales was often not practical due to a lack of domain-specific knowledge, expertise and resources. While community values should inform hygiene policy, regional or national stakeholders should lead its development and support local adaptation. This is particularly important given that effective hygiene depends not only on individual behaviors, but also on the availability and accessibility of enabling infrastructure, services, sustained funding, and supportive education and behavior-change programs, which typically require coordination and investment at regional or national levels. Education and awareness among the general public and decision makers were identified as policy enablers. Participants recommended integrating OH and hygiene principles into early education to build foundational knowledge and public support for future policy initiatives. This aligns with broader education for policy approaches that aim to strengthen policy literacy, increase risk perception and empower communities to advocate for health protective policies, and has been previously discussed for OH in the context of school and graduate-level education [91, 92].

Finally, conference participants recommended embedding hygiene-OH goals into existing policy frameworks rather than creating new ones. Examples include adapting food safety regulations (e.g., Codex standards [93]), environmental public health protocols, or animal health guidelines to reflect OH priorities. Leveraging existing policy mechanisms can improve efficiency, uptake and sustainability while avoiding duplication.

## Conclusions and recommendations

This perspective article synthesizes insights and reflections from three international science conference sessions on current gaps and priorities for strengthening alignment between hygiene and OH to protect human, animal and ecosystem health. The conference sessions engaged researchers, practitioners, policymakers and implementers from across sectors and geographies, providing a rare opportunity to compare perspectives and discuss key factors that should inform a future, integrated agenda.

Although the consultations were wide-ranging, participants were predominantly from WASH and human health backgrounds, with limited representation from food systems and ecosystem health. While the authors of this perspective article added their expertise in One Health (all authors), veterinary health (FG), and conservation (HL), the lack of ecosystem health expertise likely constrained the depth of environmental considerations in the discussions, though participants consistently emphasized the need for greater inclusion and more nuanced conceptualization of environmental and ecosystem health. Likewise, the authors’ and participants’ backgrounds likely biased discussions towards infectious disease and AMR; discussions around respiratory, vector-borne, direct contact, and other transmission pathways were notably absent. Nonetheless, the consultations engaged a diverse set of perspectives, and the gaps identified here mark clear priorities for broadening future engagement.

Hygiene and OH research and programs should prioritize purposeful coordination between agencies and actors with expertise in environmental hygiene, ecosystem health and sustainability when monitoring disease risks and designing interventions. A less anthropocentric or zoocentric approach that recognizes the ecosystem’s role in health will inherently lead to identification of more holistic hygiene programs that provide broad multispecies benefits.

From our synthesis and reflections, we present six cross-cutting recommendations for strengthening hygiene and OH alignment, focused on the environmental dimension (1), transdisciplinary research (2), linked surveillance (3), locally co-designed interventions (4), policy and regulatory integration (5), and AMR prevention as a practical entry point (6). A summary of the key gaps, implications and priority areas for aligning hygiene and One Health outlined in this perspective is provided in Table 2.

**Table 2:** Synthesis of key gaps, implications and priority actions for aligning hygiene and One Health

Domain	Identified gap	Relevance for hygiene and One Health	Priority areas for action
Evidence gaps and priorities for research	Hygiene and OH research focuses predominantly on human–animal interactions (while often disregarding benefits of these interactions), with limited consideration of the environmental / ecosystem dimension and poor integration across datasets	Limits understanding of environment-mediated transmission pathways, multispecies risks and cumulative exposures, constraining the design of effective hygiene interventions across the human–animal–environment interface	Study disease transmission across multiple species and whole ecosystems; integrate environmental sampling with human and animal data; assess costs and benefits of human–animal proximity; strengthen evidence on hygiene’s role in AMR across food systems
Methodological gaps	Lack of standardized, scalable methods for measuring fecal contamination, exposure and hygiene behaviors across human, animal and environmental settings; data systems are fragmented; need for new integrated transdisciplinary study designs and analytical tools for multi-source data collection and analysis	Prevents comparability across sectors and settings and limits the ability to link environmental contamination, behaviors and health outcomes within hygiene-OH studies	Develop standardized fecal contamination assessment tools usable in diverse settings; expand affordable diagnostics for multi-pathogen surveillance; improve exposure and behavior measurement through triangulation; harmonize data standards and platforms
Intervention development and evaluation	Hygiene interventions do not sufficiently engage local and cross-sectoral stakeholders, are human-focused and evaluated using narrow, sector-specific outcomes	Misses animal and ecosystem health, limits effectiveness and sustainability, and underestimates co-benefits across OH domains	Engage local stakeholders in cross-sectoral intervention design; balance infrastructure and behavior change; adapt existing interventions to include animal and ecosystem components; incorporate ecosystem-level actions such as pest and vegetation control; broaden evaluation to include animal, environmental and non-health outcomes
Implementation strategies	Need for context-specific, scalable implementation grounded in systems thinking; interventions often fail without local adaptation and engagement; cross-border risks require regional coordination; global coordination can leverage preparedness infrastructure	Without adaptation/local ownership and multi-scale coordination, hygiene-OH interventions are less likely to be effective or sustainable across settings	Tailor interventions to local contexts; strengthen community engagement and local decision-making; address cross-border risks through regional surveillance and coordination; leverage existing preparedness and surveillance infrastructure
Policy Development	Hygiene-related policies, standards and enforcement are fragmented across sectors and scales (e.g. regional and national), with weak alignment between human, animal and ecosystem health priorities; hygiene-OH goals are not embedded into existing frameworks; little work to build public awareness	Undermines coherent hygiene-OH action, particularly for AMR prevention and zoonotic disease control	Reduce policy fragmentation; harmonize standards and enforcement across sectors; align national and regional policies; embed hygiene-OH goals into existing frameworks (e.g. AMR plans, food safety standards); support local adaptation through higher-level policy leadership

### 1. Strengthen the environmental dimension across hygiene and One Health

The environment provides an important opportunity for closer alignment between hygiene and One Health. Hygiene programs have traditionally focused on protecting the health of select human, animal, or plant species from disease, while environmental transmission pathways, sustainability and ecosystem health remain comparatively underrepresented within OH research, governance and implementation. In a recent review of OH platforms across low- and middle-income countries, roughly two-thirds included representation from the environmental sector and in many the environmental sector assumed a subordinate role [94]. In a systematic scoping review using the Quadripartite One Health Joint Plan of Action, none of the reviewed One Health studies involved the human-environment or animal-environment sectors [95]. This limits our ability to understand and address risks arising through shared environments, including disease transmission, AMR and ecosystem degradation. Without systematically incorporating environmental dimensions, opportunities for upstream prevention across human, animal and environmental systems may be missed. Bringing hygiene and OH approaches together can broaden hygiene programs to consider impacts on animals and ecosystems, while providing practical hygiene approaches for preventing contamination and reducing exposure across shared environments.

Opportunities include ensuring environmental expertise in hygiene and One Health teams and at an institutional level (for example through formal environmental representation in national OH coordination bodies), measuring contamination of water, soil, waste and shared environments alongside human and animal health outcomes in research studies and routine surveillance; considering ecosystem impacts when designing and evaluating hygiene interventions; and aligning water and waste management standards with zoonotic disease and AMR prevention. As an example, in response to highly pathogenic avian influenza (HPAI), Brazil successfully launched an integrated response involving environmental, agricultural, and human health institutions to conduct surveillance on domestic and wild birds, biosecurity checkpoints and disinfection stations for vehicles transporting birds, coordination of sample processing, and sharing of resources [96]. Strengthening these links through existing coordination, surveillance and regulatory systems, such as AMR NAPs and zoonotic disease surveillance systems, could provide a practical route to closer hygiene–One Health alignment around shared environmental risks, without creating parallel structures.

### 2. Strengthen transdisciplinary research on hygiene-related transmission across human, animal and environmental systems

Understanding hygiene-related transmission across human, animal and environmental systems requires collaboration across disciplines that bring different perspectives on pathogens, exposure, behavior and health. Research should combine expertise and methods from fields such as epidemiology, microbiology, veterinary and animal health, environmental science, social science and behavioral research within the same study, rather than studying individual components of transmission in isolation. This includes using eco-epidemiological studies that integrate environmental sampling with human and animal health, behavior and exposure data; developing standardized tools for measuring contamination and exposure, and affordable diagnostics suitable for multispecies pathogen surveillance; and using longitudinal and mixed-methods study designs to understand when, where and how transmission occurs. Bringing these disciplines and data together can provide a stronger basis for identifying and evaluating practical hygiene interventions across the human–animal–environment interface.

### 3. Link hygiene-related surveillance across human, animal and environmental sectors using common measures and shared data

Human, animal and environmental agencies often collect hygiene-related information separately, using different indicators, sampling protocols and data systems. This limits the ability to identify shared risks, compare findings and coordinate action across sectors. National surveillance agencies, working with public health, veterinary, food safety and environmental agencies, could address this by agreeing common or comparable hygiene indicators, harmonizing sampling and laboratory protocols where appropriate, and establishing mechanisms for sharing or jointly analyzing data. The example shared in Recommendation 1 of Brazil’s response to HPAI is an instance of successful interoperable surveillance and hygiene-related control measures implemented through existing infrastructure [96]. Priority should be given to practical, low-cost measures that can be incorporated into existing systems, such as AMR surveillance networks, food safety monitoring and environmental water quality testing, rather than establishing parallel surveillance systems. For example, routinely collected measures of hygiene infrastructure (such as the presence of a handwashing facility with soap and water), environmental contamination or pathogen detection could be aligned across relevant systems to help identify where interventions are needed. At the global level, the Quadripartite agencies could support this by developing guidance on a core set of comparable hygiene indicators and associated metadata, promoting interoperable data standards, and providing frameworks for data sharing and joint analysis across human, animal and environmental surveillance, while allowing data to remain within existing sectoral systems.

### 4. Co-design practical hygiene interventions with local stakeholders for high-risk human—animal—environment interfaces

Many important human-animal-environment interactions occur in informal or small-scale settings that are poorly reached by formal regulation or standard health programs, including smallholder farms, traditional animal markets and peri-urban water and waste systems. These settings are important opportunities for upstream prevention through localized hygiene interventions (i.e., interventions tailored to a specific community or exposure setting). Local authorities and program implementers should work with communities, public health and veterinary services, agricultural extension services and other relevant local actors to identify and co-design context-specific hygiene interventions that target the human–animal–environment interface while reflecting local practices, infrastructure and priorities. Examples include improving hygiene practices and protocols, including waste disposal systems, water protection measures, and food vendor and market hygiene protocols. Interventions should not only focus on behavior change but also ensure that the infrastructure and services required are available to make safer practices possible, rather than placing responsibility primarily on individuals and households. Evaluation should also consider impacts across human, animal, and environmental health, alongside locally important benefits, costs, and trade-offs. Strengthening collaboration between community stakeholders and local authorities can improve feasibility, equity, cultural appropriateness, and long-term sustainability of interventions.

### 5. Integrate hygiene standards into existing One Health policy and regulatory frameworks

Hygiene is addressed to varying degrees across public health, food safety, animal health and environmental policy, but its inclusion is often uneven and fragmented across sectors and levels of governance, limiting coordinated risk management and leading to inconsistent implementation of regulation. Governments and regulatory agencies should therefore strengthen coordination and alignment between existing policies across sectors. This could include harmonizing hygiene standards across food production, animal husbandry, healthcare and environmental management; clarifying responsibilities for hygiene-related risks that span sectoral mandates; and adapting existing frameworks, such as food safety standards, environmental public health protocols and animal health guidelines, to better address linked human, animal and environmental risks. Regional and global organizations can support this by promoting compatible standards and guidance across countries, while national authorities should support local adaptation according to infrastructure, resources and context. Rather than creating parallel systems, existing surveillance systems, pandemic preparedness networks, laboratory platforms, and cross-sectoral collaboration platforms can be leveraged to monitor hygiene-related risk across sectors and inform coordinated prevention and response.

### 6. Use AMR prevention as a practical starting point for stronger hygiene–One Health alignment

AMR provides a particularly clear opportunity to bring hygiene and One Health together across research, policy and practice [97, 98]. Hygiene already contributes to AMR prevention through infection prevention and control, biosecurity, animal husbandry and food production, but these activities are not consistently connected across human, animal and environmental systems. Governments and agencies responsible for AMR could strengthen these links through existing One Health National Action Plans, including greater attention to hygiene across healthcare, food value chains, animal production and environmental pathways such as waste and water management. Existing AMR surveillance, laboratory and program infrastructure can also support coordinated action across sectors. At the same time, researchers should evaluate the effectiveness, feasibility and incentives needed for hygiene interventions to reduce infection and antimicrobial use, particularly in livestock production and food systems. AMR prevention can therefore serve as a practical example to demonstrate how coordinated hygiene interventions can address shared risks and transmission pathways across human, animal and environmental systems.

Together, these recommendations provide a practical agenda for moving from fragmented, sector-specific efforts toward a coordinated, cross-sectoral hygiene-OH approach. Achieving this will require deliberate inclusion of environmental expertise, stronger coordination of systems and policies, and local ownership of solutions. Closer alignment of hygiene and One Health can support more coordinated, upstream prevention across human, animal and environmental systems.

## Data Availability

No datasets were generated or analysed during the current study.

## Abbreviations

OH: One Health
OHJPA: One Health Joint Plan of Action
AMR: antimicrobial resistance
WASH: water, sanitation and hygiene
LMICs: low- and middle-income countries
SDGs: Sustainable Development Goals
UNC: University of North Carolina Water and Health Conference
GHS: Global Hygiene Symposium
ASTMH: American Society of Tropical Medicine and Hygiene annual meeting
